# Comparison of muscle activity of the lower limbs while running on different treadmill models

**DOI:** 10.3389/fnhum.2024.1341772

**Published:** 2024-04-04

**Authors:** Christina Kaltenbach, Albert Gollhofer, Benno M. Nigg, Michael J. Asmussen

**Affiliations:** ^1^Department of Sport and Sports Science, University of Freiburg, Freiburg, Germany; ^2^Faculty of Kinesiology, University of Calgary, Calgary, AB, Canada; ^3^Department of Kinesiology, Faculty of Education, Vancouver Island University, Nanaimo, BC, Canada

**Keywords:** treadmill, overground running, muscle activity, electromyography, running, surface mechanical properties

## Abstract

Treadmill running is a common method of exercise and to study human locomotion. Research has examined the kinematics and kinetics of overground and treadmill running, but there has been less focus on the levels of muscle activity during treadmill running. We investigated if muscle activity is different while running overground compared to running on a variety of treadmills. A total of 11 healthy individuals ran at 3 speeds (2.6, 3.6, 4.5 m/s) under 4 different running conditions (3 treadmills, overground). The three treadmills included a typical home exercise treadmill, a midsize commercial research treadmill, and a large, instrumented research treadmill. Surface EMG of the tibialis anterior (TA), gastrocnemius medialis (GM), rectus femoris (RF) and biceps femoris (BF) muscles were measured for each running condition. The integrated EMG was computed for each running condition for the stance and swing phase, as well as 100 ms before and after the heel-strike. Friedman analysis revealed significant effects during the stance phase for GM and RF at all speeds, such that muscle activation was lower on the treadmills relative to overground. During the stance phase at faster speeds, the muscle activity was higher for the TA and lower for the BF while running on the different treadmills compared to overground running. Before heel-strike, the TA was significantly less active during treadmill compared to overground running at 2.6 m/s and the RF showed significantly higher activity at 3.6 m/s and 4.5 m/s while running on the different treadmills. Summarizing, differences were mainly observed between the different treadmill conditions relative to overground running. Muscle activation differences between the different treadmill conditions were observed at faster running speeds for RF during the pre-heel-strike phase only. Different types of treadmills with different mechanical properties affects the muscle activity during stance phase as well as in preparation to heel-strike. Additionally, the muscle activity is greater during overground compared to treadmill running during the stance phase for the GM, BF, and RF.

## 1 Introduction

Bipedal human locomotion is important for day to day function. This task can be studied in a laboratory setting using two main methods. One method involves overground walking and running, whereby a person moves over a surface as they would in their everyday lives. Another method uses a treadmill, allowing for a person to remain relatively stationary mimicking the analogous overground forms of locomotion. Because of a variety of factors, such as lab space, repeatability, speed control, or convenience, locomotion is typically studied using a treadmill (Nigg et al., 1995). Research to date comparing overground to treadmill running has shown contrasting results such that some researchers have argued that treadmill running may be quite different than overground running (Nelson et al., 1972; Nigg et al., 1995; Alton et al., 1998; Wank et al., 1998; Wang et al., 2014), while others have argued that these two methods of running are similar (Kram and Powell, 1989; Dierick et al., 2004; Riley et al., 2007; Lee and Hidler, 2008).

Based on previous studies in the area, comparisons of the type of treadmill running has examined the traditional kinematics and kinetics of running, but fewer studies have focused on muscle activity comparisons. Once again, in this area of muscle activity research, the evidence shows conflicting results with some researchers arguing that muscle activity is different between these different running conditions (Wank et al., 1998; Baur et al., 2007; Lee and Hidler, 2008), while others suggest that it is not (Murray et al., 1985; Arsenault et al., 1986). Researchers have argued that when there are differences in muscle activity between running surface conditions, these to changes in muscle activity are attributed to differences in running kinematics (e.g., shorter steps on treadmills; Arsenault et al., 1986). One factor that is often overlooked, is the treadmill surface type and how it may influence muscle activity during running. No studies, however, attribute the changes in muscle activity to the type of treadmill that the participants ran on.

It is well known that technical specifications (i.e., material, thickness, temperature, energy storage and return, and performance) and sport functional properties (i.e., stiffness, friction, traction, compliance, and force reduction) of the running surface influences running performance (Nigg and Yeadon, 1987; Dixon et al., 2005), such that a higher activation in the tibialis anterior, and during walking, the soleus muscles was found on a compliant surface when compared to a more rigid surface (MacLellan and Patla, 2006). Researchers have also shown that runners adjust their leg stiffness when running on a surface with different mechanical properties and this change can happen within a single step (Ferris et al., 1999). Further to this point, researchers have shown that the mechanical properties of treadmill surfaces are much different than standard overground sport surfaces and this difference, although perceptively subtle, may affect a person's running performance and their injury risk (Colino et al., 2020). The specific main variations in mechanical properties of treadmills are the surface stiffness (Van Hooren et al., 2020) and the stiffness and damping characteristics of the treadmill frame (Asmussen et al., 2019), which can vary across models.

Although not fully resolved, there are likely biomechanical differences between treadmill running and overground running (Van Hooren et al., 2020). If true, it is not only important to understand how overground running compares to treadmill running, but it is equally important to understand how a person's biomechanics change when running on different treadmill models. From a translation standpoint, this topic is even more relevant considering that treadmills are widely used by the general public as a piece of exercise equipment. The majority of research to date comparing overground and treadmill running has been conducted using highly expensive instrumented treadmills (Riley et al., 2007). This methodological approach could affect the generalizability of research findings because these treadmills are typically used in research studies, but not by the general public to exercise. Instead, the majority of the population run on treadmills that are not as expensive, as stiff, and as well designed as the treadmills in a laboratory setting. Thus, it is not yet understood how running on these different treadmills at different speeds affects the neuromuscular system controlling locomotion (Nigg et al., 1995).

Researchers arguing in favor of treadmill running being different that overground running have suggested that people must compensate for the mechanical differences of running on a treadmill by stabilization of the joint (Ford et al., 2008) through a simultaneous co-activation of the muscles acting around the specific joints (Kellis et al., 2003) as a means to maintain postural stability on the treadmill (Baur et al., 2007; Oliveira et al., 2016). Muscle activity might also be altered when running on a treadmill because it has been reported that running on treadmills requires less propulsive force forward as the belt moves the legs below the body, while in overground running a forward progression of the trunk is necessary to move the body forward (Baur et al., 2007; Van Hooren et al., 2020). In favor of treadmill running reducing muscle activity, the belt moving under the person's body could create a mechanical “pulling” of the leg that would reduce activity from muscles that control hip extension and knee flexion, depending on the point in the stance phase. Treadmill running could also increase muscle activity to stabilize the joint that could be a factor of the narrow belt surface or belt slip that occurs during initial foot contact. If muscle activity is drastically altered across different treadmills, it could indicate that muscles are producing different levels of force when using different treadmill models and has application to sport performance, potential overuse injury risk (Howard et al., 2018), and the generalizability of previous research findings.

Therefore, the primary purpose of the study was to determine if muscle activity was altered when running on different types of treadmills and if muscle activity differed between overground and treadmill running. It was hypothesized that muscle activity would be different between the treadmill conditions and overground running. It was further hypothesized that muscle activity would be different between the different treadmill conditions.

## 2 Methods

Eleven recreational runners (7 males and 4 females, mean ± SD values: age: 26 ± 2 yrs., body mass: 70.3 ± 8.7 kg, height: 174.0 ± 7.4 cm) who ran 1–4 times/week provided written, informed consent to participate in this study. This participant number was based on a power analysis using previous research summarizing muscle activity differences between treadmill and overground running with inputs of α = 0.01, (1-β) = 0.95 and *dz* = 1.63 (Matsas et al., 2000). The study was approved by the University of Calgary's Conjoint Health Research Ethics Board policy on research using human subjects.

### 2.1 Testing protocol

The participants ran at three different speeds: 2.7, 3.6, and 4.5 m/s for a minimum of 30 s in their own running shoes on three different treadmills and an overground running condition. The order of the conditions was randomized. Five trials of overground running (30 m runway) at the same speed setting as participants ran on the treadmill (2.7, 3.6, and 4.5 m/s) were completed. The running speed was collected with timing lights for the overground condition. Participants performed familiarization trials for treadmill and overground running before conducting the experimental trials. The familiarization trial also included a warm up because it has previously been shown that short warm ups are enough not to enhance intermediate performance (Van den Tillaar et al., 2017) and a minimum of 6 min of warm-up was implemented according to previous research (Matsas et al., 2000). All participants were rear-foot strike runners.

### 2.2 Instrumentation

For the study, participants ran on three different treadmills and one overground running condition with the following specifications:

(i) Quinton Q65 (Quinton Instruments Co., Seattle) a mid-size (1.4 × 0.5 m) commercially available research treadmill. The speed range is 1.0 – 14 mph and with a 3.3 kW motor power.
(ii) Healthrider H20T (ICON Health & Fitness Ltd., Logan) a less expensive, small-size (1.27 × 0.41 m) home exercise treadmill. The treadmill also included a cushioning system between the belt and the frame. The speed range is 1.0 – 10 mph with a 1.5 kW motor power.
(iii) Bertec (Bertec Corporation, Columbus, OH, fully instrumented treadmill with incline feature) an expensive, large (1.75 × 0.5 m) research treadmill with force plates (FP4550-08-TM, 1000 Hz) embedded within the treadmill structure to record the force applied on each belt. The treadmill was clamped down to increase the stiffness and prevent excessive vibrations when the foot is in contact with the treadmill. The speed range is 1.0–14.5 mph with a 2.6 kW motor power.
(iv) Overground running was conducted on a sport surface in a running lane of 30 m with participants landing on a rigid force plate (Kistler Instrumente AG, Switzerland, 2400 Hz, Type 9287) embedded in the floor. The force plate was not covered by sport surface, such that each participant contacted the force plate directly with their foot.

The timing lights were placed at 1.90 meters from each edge of the force plate and marked with tape on the floor to make sure that distance was the same for each subject. The running trial was included when the participant landed on the force plate with the dominant leg and foot and the speed was in the correct range of ±0.5 m/s per speed.

To make accurate comparisons between treadmills, we ensured the belt speed was constant over all the speed conditions by measuring the belt speed variations of all treadmills over all speeds before choosing the three speed settings that were the most identical across treadmill conditions. The belt speed variation of each treadmill was measured by placing a fixed camera on the treadmill, placing tape on the belt and on the frame. We measured the speed of the belt by knowing the length of the belt and the length of time for the belt to complete a full loop.

Surface electromyography (sEMG; Biovision, Germany, 2400 Hz) recordings, using a bipolar montage, was conducted by placing electrodes on the skin overlying the muscle belly of the tibialis anterior (TA), gastrocnemius medialis (GM), rectus femoris (RF) and biceps femoris (BF) of the dominant leg using the SENIAM guidelines (Hermens et al., 2000). The electrodes were placed after removal of hair, abrasion of the skin with abrasive tape and cleaned with alcohol tissue. A ground electrode was placed on the lateral condyle of the knee. For each recorded muscle, the persons' maximum voluntary contraction was obtained by having the experimenter or a rigid structure provide resistance whilst the participant performed the maximal contraction. While seated, the participant performed foot dorsiflexion against resistance by the experimenter for the tibialis anterior, foot plantarflexion was performed against resistance provided by a rigid structure for the gastrocnemius medialis, leg extension was performed against resistance by a rigid structure for the rectus femoris, and leg flexion was performed with resistance provided by the experimenter for the biceps femoris.

The bipolar EMG signal was amplified with a differential amplifier (Biovision, Germany; amplification 1000) and sent to an analog-to-digital (A/D) converter. A single 1D accelerometer (Biovision, Germany, 2400 Hz) attached to the heel of the dominant leg and force plates (Kistler Instrumente AG, Switzerland, 2400 Hz, Type 9287) was used to synchronize the data acquisition systems.

### 2.3 Data processing

Data processing of the EMG was completed with custom developed scripts using MATLAB (R2016A, Mathworks, USA) and conducted by taking the wavelet transformation of the EMG signal (von Tscharner et al., 2003). Using the wavelet transformed EMG, the signal was analyzed as the square of the amplitude of the EMG signal contained within a particular frequency band (Wakeling et al., 2003). To be consistent across the conditions, five gait cycles for the treadmill conditions and five for the overground condition were used for the EMG analysis. To calculate the gait cycle, we used the same set up as described in Asmussen et al. (2019) such that the force, accelerometer, and EMG signals were all time-aligned. The Quinton and the Healthrider treadmill were placed on top of two force plates (Kistler Instrumente AG, Switzerland, 2400 Hz, Type 9287): one in the front under the support legs and one in the back under the support legs of the treadmills. Specifically, to time-align the data, a synchronization event was introduced across all data collection systems, whereby participants used their heel to strike the frame of the treadmill three times at the beginning of a trial and two times at the end of each trial. For the overground running condition, the participants started the trial at the force platform where they struck the force plate three times with the dominant foot, found their defined spot to start, and completed the running trial. After contacting the force plate with the dominant foot, the subjects went back to the force plate and struck it again two times with their dominant foot to end the trial. These synchronization events created peaks in the accelerometer data and force platform data - the EMG data was collected with the same system as the accelerometer and therefore, was synchronized with these events. After these events, the gait cycle was divided into two phases in stance-phase and swing-phase. Stance-phase was determined from initial contact of the heel (first crossing of a threshold of 50 N) until toe-off (the second crossing of a 50N threshold). The swing phase was defined from toe-off until heel-strike of the same foot. The signal was then normalized to a 100% of the gait phase.

We performed the EMG analysis using two different methods to separate parts of the gait cycle. In the first method, we used a more traditional approach of performing the analysis separated into swing phase and stance phase. Due to differences in the mechanical properties of the treadmill surfaces at heel-strike (Asmussen et al., 2019) and given that gait patterns change dependent on the surface conditions (Ferris et al., 1999), our second method evaluated the muscle activity of gait separated into events before and after heel-strike to understand the changes in EMG while participants were preparing to contact the surface and the changes immediately after surface contact. Further to this point, it has been proposed that the vertical impact peak force may cause alterations in the muscle activity (Wakeling et al., 2003) and for this reason, a short time window was used to determine the muscle activity surrounding heel-strike in comparison to the impact peak of the ground reaction force. For instance, von Tscharner et al. (2003) determined that the main EMG activity of the tibialis anterior occurred in the last 100 ms before and 100 ms after heel-strike when analyzing muscle intensity. To perform this additional second method of analysis, the activation of the EMG signal was calculated from a 100 ms time window before and after the heel strike. All data were normalized to the person's maximum voluntary contraction (%MVC) and the mean integrated EMG was calculated (trapezoidal integration method).

Statistical analysis was completed with R Core Team (2019). Due to missing data, two subjects had to be excluded from the analysis. Nine subjects remained after exclusion. The nonparametric Friedman test among repeated measure was used for differences in the running condition (3 treadmill conditions; overground). Because muscle activity would be larger with increases in running speed (Kyröläinen et al., 2005) and the main purpose of the study was to determine differences across the running conditions, we restricted the analysis to the Friedman test for each speed setting (2.7, 3.6, 4.5 m/s) and for each muscle (TA, GM, RF, BF). In other words, we were not concerned with the interaction between running condition and speed or running condition and muscle. All tests performed were evaluated against a significance level of α = 0.05 and effect size with Kendall's *W* with 0.1 < 0.3, 0.3< 0.5, 0.5< indicating a small, medium, and large effect size, respectively. If a significant effect was found, the *post-hoc* non-parametric pairwise comparison Durbin-Conover analysis with the Bonferroni *p*-adjustment was conducted.

### 2.4 Experimental modal analysis

To determine the dynamic behavior of each treadmill structure, an experimental modal analysis was performed on the Quinton and the Healthrider treadmills. This experimental modal analysis can assess the natural frequency and the mode shape of a structure, which in this case is each treadmill. Accelerometers were placed at each corner of the treadmill and along points in between. An impact modal test was performed. For this modal test, the input force to the treadmill was applied by an instrumented force hammer (PCB 2222), which allows the experimenter to know the precise force used to excite the structure. The instrumented hammer then records the impulse response applied to the treadmill. The oscillations of the treadmill were measured by the accelerometers (B&K 4508B). This measurement from the accelerometers was the output or response to the excitation provided by the instrumented hammer. With these two measures, the experimenter can determine the frequency response function, which will give the natural frequency, damping, and mode shape.

## 3 Results

### 3.1 Stance phase and swing phase– method 1

**Figure 1:**
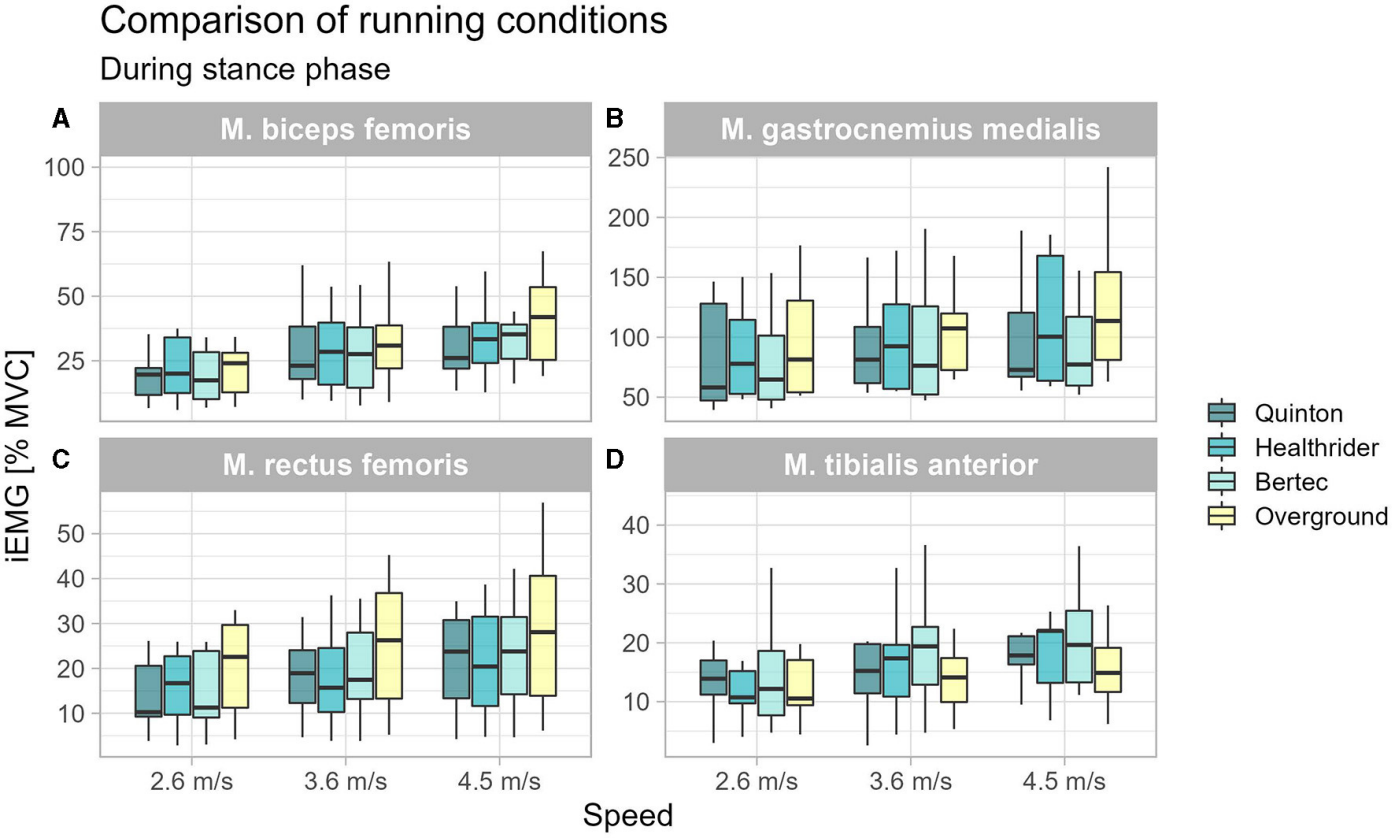
Boxplot of the mean of the integrated EMG of the running conditions over the different speed settings during stance phase for the biceps femoris **(A)**, gastrocnemius medialis **(B)**, rectus femoris **(C)**, and tibialis anterior **(D)**.

During the stance phase, the tibialis anterior activity was significantly altered between the running conditions at 3.6 m/s (*x*^2^_Friedman_ = 8.87, *p* = 0.031, W_Kendall_ = 0.33, 95% CI [0.16, 0.60]) and 4.5 m/s (*x*^2^_Friedman_ = 10.73, *p* = 0.013, W_Kendall_ = 0.40, 95% CI [0.22, 0.83]). The *post-hoc* analysis revealed that there was no difference between the treadmill conditions, but there were differences between the Bertec treadmill relative to overground running at 3.6 m/s (*p* = 0.026) and 4.5 m/s (*p* = 0.006) and Healthrider treadmill vs. the overground condition at the running speed of 4.5 m/s (*p* = 0.049). A significant effect for the biceps femoris muscle activity during stance phase was found at 4.5 m/s (*x*^2^_Friedman_ = 14.73, *p* = 0.002, W_Kendall_ = 0.55, 95% CI [0.29, 0.83]). The *post-hoc* analysis indicated no differences between the treadmills, however, there was a significantly higher activation during overground running compared to Quinton treadmill (*p* = 0.001) and the Healthrider treadmill (*p* < 0.001). The Friedman analysis revealed significant effects for the gastrocnemius medialis and for the rectus femoris across all speed conditions (see Table 1). The *post-hoc* analysis indicated that these significant differences were between the treadmill conditions and overground running, but not between the different treadmill conditions for the gastrocnemius and rectus femoris muscles (see Table 1). The increased muscle activity of gastrocnemius medialis, biceps femoris and rectus femoris during overground running compared to treadmill running is shown in Figure 1.

**Table 1:** The results of the statistical analysis of the Friedman rank sum test and the associated *p-*value of stance phase for each of the four muscles across the different treadmill conditions over the three speed settings.

**Stance phase**		**Friedman test**	***p*-value**	**W_Kendal_**	**95 % Confidence interval**		**Durbin- Conover pairwise comparison**
**Muscle**	**Speed**	**(***n*_pairs_ = **9)**			**Lower bound**	**Upper bound**	
**TA**	2.6 m/s	*x*^2^ = 4.33	0.228	0.16	0.06	0.59	
	3.6 m/s	*x*^2^ = 8.87	**0.031** ^*^	0.33	0.16	0.6	BE - OG *p =* 0.026
	4.5 m/s	*x*^2^ = 10.73	**0.013** ^*^	0.4	0.22	0.83	HR - OG *p =* 0.049
							BE - OG *p =* 0.006
**RF**	2.6 m/s	*x*^2^ = 14.20	**0.003** ^**^	0.53	0.45	0.79	QU - OG *p =* 0.001
							HR - OG *p =* 0.002
							BE - OG *p =* 0.006
	3.6 m/s	*x*^2^ = 10.73	**0.013** ^*^	0.4	0.21	0.7	QU - OG *p =* 0.017
							HR - OG p = 0.01
	4.5 m/s	*x*^2^ = 10.20	**0.017** ^*^	0.38	0.24	0.74	QU - OG *p =* 0.007
**BF**	2.6 m/s	*x*^2^ = 3.80	0.284	0.14	0.05	0.53	
	3.6 m/s	*x*^2^ = 5.13	0.162	0.19	0.07	0.62	
	4.5 m/s	*x*^2^ = 14.73	**0.002** ^**^	0.55	0.29	0.83	QU - OG *p =* 0.001
							HR - OG p < 0.001
**GM**	2.6 m/s	*x*^2^ = 13.53	**0.004** ^**^	0.5	0.31	0.8	QU - OG *p =* 0.001
							BE - OG *p =* 0.004
	3.6 m/s	*x*^2^ = 12.07	**0.007** ^**^	0.45	0.25	0.74	QU - OG *p =* 0.012
							HR - OG *p =* 0.021
							BE - OG *p =* 0.004
	4.5 m/s	*x*^2^ = 12.33	**0.006** ^**^	0.46	0.31	0.7	QU - OG *p =* 0.003
							BE - OG *p =* 0.006

The tests with significant differences are marked bold and significant *post-hoc* comparisons are presented. x^2^, Friedman rank sum test; W_*Kendall*_, Kendall's coefficient of concordance; CI_95%_, 95 % Confidence Interval; n_*pair*_, number of observations; TA, M. tibialis anterior; GM, M. gastrocnemius medialis; BG, M. biceps femoris; RF, M. rectus femoris; QU, Quinton treadmill; HR, Healthrider treadmill; BE, Bertec treadmill; OG, overground. ^*^p ≤ 0.05, ^**^p ≤ 0.01.

**Figure 2:**
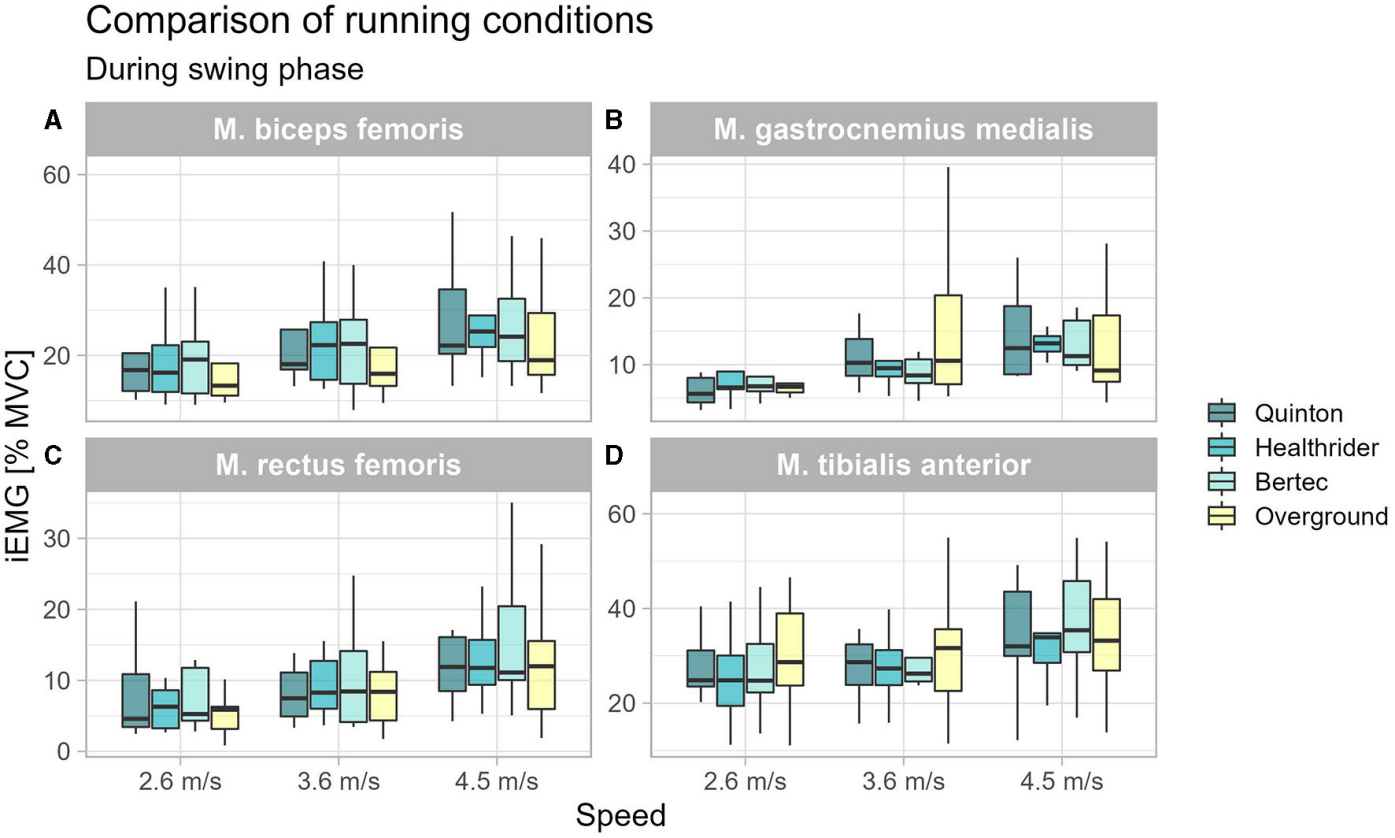
Boxplot of the mean of the integrated EMG of the running conditions over the different speed settings during swing phase for the biceps femoris **(A)**, gastrocnemius medialis **(B)**, rectus femoris **(C)**, and tibialis anterior **(D)**.

During swing phase only, the biceps femoris was altered during running at 3.6 m/s (*x*^2^_Friedman_ = 11.40, *p* = 0.010, W_Kendall_ = 0.42, 95% CI [0.21, 0.84]) between the running conditions (see Table 2). Further nonparametric *post-hoc* analysis can be seen in Figure 2, showing that the differences were not between the treadmill conditions, but instead, the Quinton treadmill (*p* = 0.008) and Bertec treadmill (*p* = 0.008) vs. overground running.

**Table 2:** The results of the statistical analysis of the Friedman rank sum test and the associated *p*-value of swing phase for each of the four muscles across the different treadmill conditions over the three speed settings.

**Swing phase**		**Friedman test (*n*_pairs_ = 9)**	***p*-value**	**W_Kendal_**	**95 % confidence interval**		**Durbin- Conover pairwise comparison**
**Muscle**	**Speed**				**Lower bound**	**Upper bound**	
TA	2.6 m/s	*x*^2^ = 4.07	0.254	0.15	0.04	0.67	
	3.6 m/s	*x*^2^ = 2.20	0.532	0.08	0.01	0.65	
	4.5 m/s	*x*^2^ = 5.13	0.162	0.19	0.04	0.61	
RF	2.6 m/s	*x*^2^ = 1.40	0.706	0.05	0.03	0.52	
	3.6 m/s	*x*^2^ = 2.87	0.413	0.11	0.01	0.58	
	4.5 m/s	*x*^2^ = 1.93	0.586	0.07	0.01	0.48	
BF	2.6 m/s	*x*^2^ = 7.00	0.072	0.26	0.11	0.59	
	3.6 m/s	*x*^2^ = 11.40	**0.010** ^**^	0.42	0.21	0.84	QU - OG *p =* 0.008 BE - OG *p =* 0.008
	4.5 m/s	*x*^2^ = 6.73	0.081	0.25	0.05	0.66	
GM	2.6 m/s	*x*^2^ = 0.60	0.896	0.02	0.01	0.38	
	3.6 m/s	*x*^2^ = 0.33	0.954	0.01	0.00	0.39	
	4.5 m/s	*x*^2^ = 4.60	0.204	0.17	0.07	0.65	

The tests with significant differences are marked bold and significant *post-hoc* comparisons are presented. x^2^, Friedman rank sum test; W_*Kendall*_, Kendall's coefficient of concordance; CI_95%_, 95 % Confidence Interval; n_*pair*_, number of observations; TA, M. tibialis anterior; GM, M. gastrocnemius medialis; BG, M. biceps femoris; RF, M. rectus femoris; QU, Quinton treadmill; HR, Healthrider treadmill; BE, Bertec treadmill; OG, overground. ^**^p ≤ 0.05.

### 3.2 Pre- and post-heel-strike–method 2

**Figure 3:**
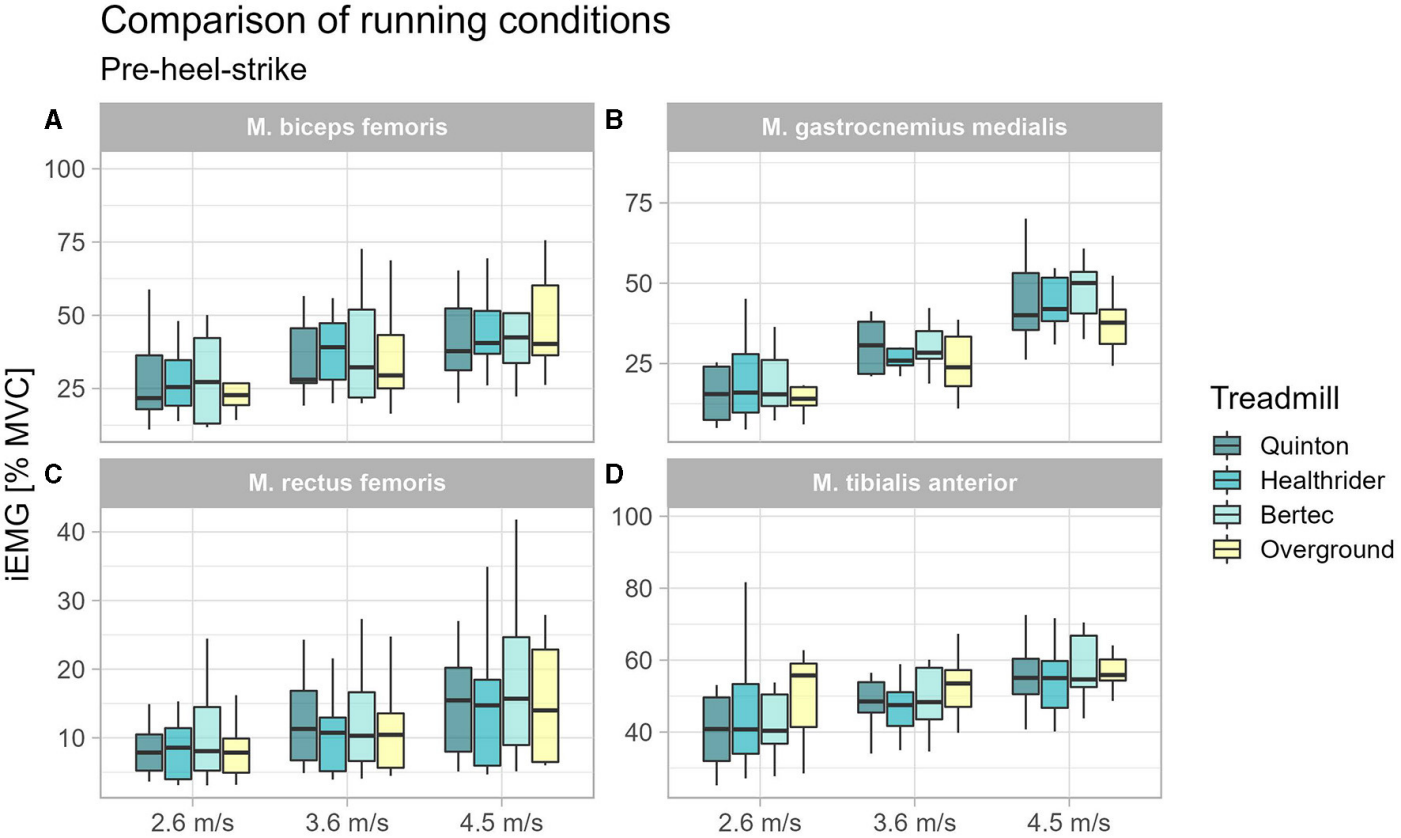
Boxplot of the mean of the integrated EMG of the running conditions over the different speed settings for the time window 100 ms before heel-strike for the biceps femoris **(A)**, gastrocnemius medialis **(B)**, rectus femoris **(C)**, and tibialis anterior **(D)**.

To understand the changes in muscle activity immediately before surface contact, we analyzed muscle activity before heel-strike. A significant effect for the tibialis anterior at 2.6 m/s (*x*^2^_Friedman_ = 11.53, *p* = 0.009, W_Kendall_ = 0.43, 95% CI [0.18, 0.83]). Significant differences were found with the *post-hoc* analysis for the tibialis anterior at 2.7 m/s between the treadmill conditions Healthrider (*p* = 0.041) and Quinton (*p* = 0.002) relative to overground running, but no differences between treadmill conditions. For the rectus femoris muscle at 3.6 m/s (*x*^2^_Friedman_ = 9.13, *p* = 0.028, W_Kendall_ = 0.34, 95% CI [0.19, 0.67]) and at 4.5 m/s (*x*^2^_Friedman_ = 11.00, *p* = 0.012, W_Kendall_ = 0.41, 95% CI [0.20, 0.77]), significant differences were found. *Post-hoc* analyses showed differences for the rectus femoris activity between the treadmill conditions, namely the Healthrider vs. Quinton treadmills (*p* = 0.015) at 3.6 m/s and between Bertec and Healthrider (*p* = 0.003) treadmills at 4.5 m/s. No differences were found for the biceps femoris and gastrocnemius medialis muscle. The results from the *post-hoc* analysis of the pre-heel-strike are presented in Table 3 and Figure 3.

**Table 3:** Mean and SE iEMG of pre-heel-strike (100 ms before heel-strike) for each of the four muscles across the different treadmill conditions over the three speed settings and the results of the statistical analysis of the Friedman rank sum test and the associated *p*-value.

**Pre-heel strike**		**Friedman test (*n*_pairs_ = 9)**	***p*-value**	**W_Kendal_**	**95 % confidence interval**		**Durbin- Conover pairwise comparison**
**Muscle**	**Speed**				**Lower bound**	**Upper bound**	
TA	2.6 m/s	***x***^2^ **=** **11.53**	**0.009** ^**^	0.43	0.18	0.83	HR - OG *p =* 0.041 QU - OG p = 0.002
	3.6 m/s	*x*^2^ = 2.73	0.435	0.10	0.04	0.46	
	4.5 m/s	*x*^2^ = 0.87	0.833	0.03	0.02	0.40	
RF	2.6 m/s	*x*^2^ = 5.13	0.162	0.19	0.06	0.66	
	3.6 m/s	*x*^2^ = 9.13	**0.028** ^*^	0.34	0.19	0.67	HR - QU *p =* 0.015
	4.5 m/s	*x*^2^ = 11.00	**0.012** ^*^	0.41	0.20	0.77	BE- HR *p =* 0.003
BF	2.6 m/s	*x*^2^ = 1.40	0.706	0.05	0.01	0.47	
	3.6 m/s	*x*^2^ = 2.20,	0.532	0.06	0.00	0.63	
	4.5 m/s	*x*^2^ = 4.33	0.228	0.16	0.08	0.45	
GM	2.6 m/s	*x*^2^ = 2.47	0.481	0.09	0.01	0.61	
	3.6 m/s	*x*^2^ = 1.93	0.586	0.07	0.02	0.52	
	4.5 m/s	*x*^2^ = 7.00	0.072	0.26	0.14	0.70	

The tests with significant differences are marked bold and significant *post-hoc* comparisons are presented. x^2^, Friedman rank sum test; W_*Kendall*_, Kendall's coefficient of concordance; CI_95%_, 95 % Confidence Interval; n_*pair*_, number of observations; TA, M. tibialis anterior; GM, M. gastrocnemius medialis; BG, M. biceps femoris; RF, M. rectus femoris; QU, Quinton treadmill; HR, Healthrider treadmill; BE, Bertec treadmill; OG, overground. ^*^p ≤ 0.05, ^**^p ≤ 0.01.

**Figure 4:**
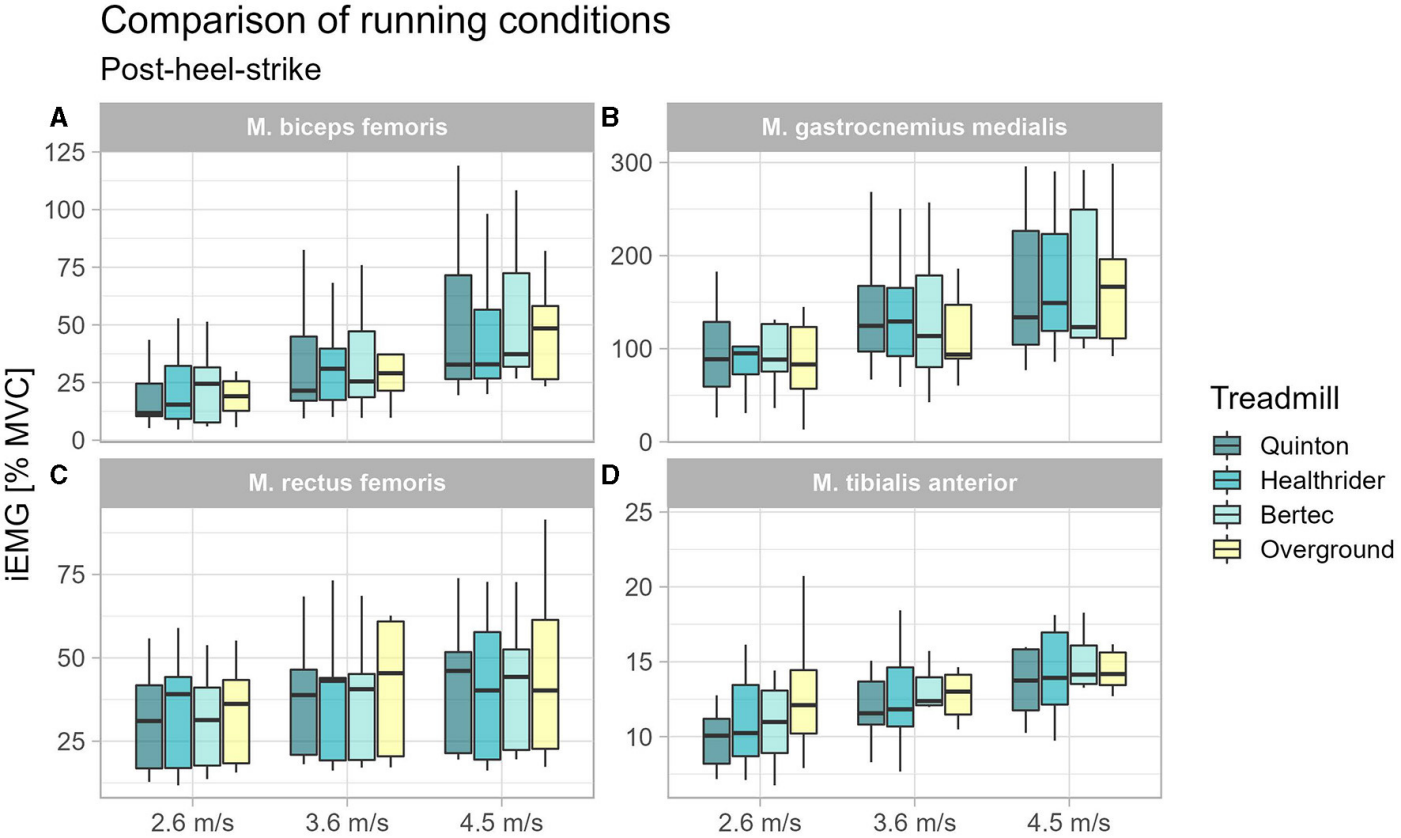
Boxplot of the mean of the integrated EMG of the running conditions over the different speed settings for the time window 100 ms after heel-strike for the biceps femoris **(A)**, gastrocnemius medialis **(B)**, rectus femoris **(C)**, and tibialis anterior **(D)**.

During post heel strike, the tibialis anterior activity was the only muscle with altered muscle activity at 2.6 m/s (*x*^2^_Friedman_ = 10.87, *p* = 0.012, W_Kendall_ = 0.40, 95% CI [0.18, 0.81]). A significant difference was found in the follow up pairwise comparison for the TA between the Quinton treadmill and overground condition (*p* = 0.003) at 2.6 m/s, but no differences between treadmill conditions. Results of the post-heel-strike are presented in Table 4 and Figure 4.

**Table 4:** Mean and SE iEMG of post-heel-strike (100 ms after heel-strike) for each of the four muscles across the different treadmill conditions over the three speed settings and the results of the statistical analysis of the Friedman rank sum test and the associated *p*-value.

**Post-heel-strike**		**Friedman test (*n*_pairs_ = 9)**	***p*-value**	**W_Kendal_**	**95 % confidence interval**		**Durbin- Conover pairwise comparison**
**Muscle**	**Speed**				**Lower bound**	**Upper bound**	
TA	2.6 m/s	*x*^2^ = 10.87	**0.012** ^*^	0.40	0.18	0.81	QU - OG *p =* 0.003
	3.6 m/s	*x*^2^ = 3.40	0.334	0.13	0.03	0.59	
	4.5 m/s	*x*^2^ = 4.60	0.204	0.17	0.07	0.57	
RF	2.6 m/s	*x*^2^ = 2.20	0.532	0.08	0.01	0.66	
	3.6 m/s	*x*^2^ = 1.40	0.706	0.05	0.02	0.49	
	4.5 m/s	*x*^2^ = 4.47	0.215	0.17	0.03	0.54	
BF	2.6 m/s	*x*^2^ = 3.27	0.352	0.12	0.02	0.60	
	3.6 m/s	*x*^2^ = 2.20	0.532	0.08	0.02	0.53	
	4.5 m/s	*x*^2^ = 3.40	0.334	0.13	0.04	0.56	
GM	2.6 m/s	*x*^2^ = 3.93	0.269	0.15	0.03	0.74	
	3.6 m/s	*x*^2^ = 3.40	0.334	0.13	0.04	0.57	
	4.5 m/s	*x*^2^ = 0.20	0.978	0.007	0.00	0.30	

The tests with significant differences are marked bold and significant *post-hoc* comparisons are presented. x^2^, Friedman rank sum test; W_*Kendall*_, Kendall's coefficient of concordance; CI_95%_, 95 % Confidence Interval; n_*pair*_, number of observations; TA, M. tibialis anterior; GM, M. gastrocnemius medialis; BG, M. biceps femoris; RF, M. rectus femoris; QU, Quinton treadmill; HR, Healthrider treadmill; BE, Bertec treadmill; OG, overground. ^*^p ≤ 0.05.

### 3.3 Experimental modal analysis

**Figure 5:**
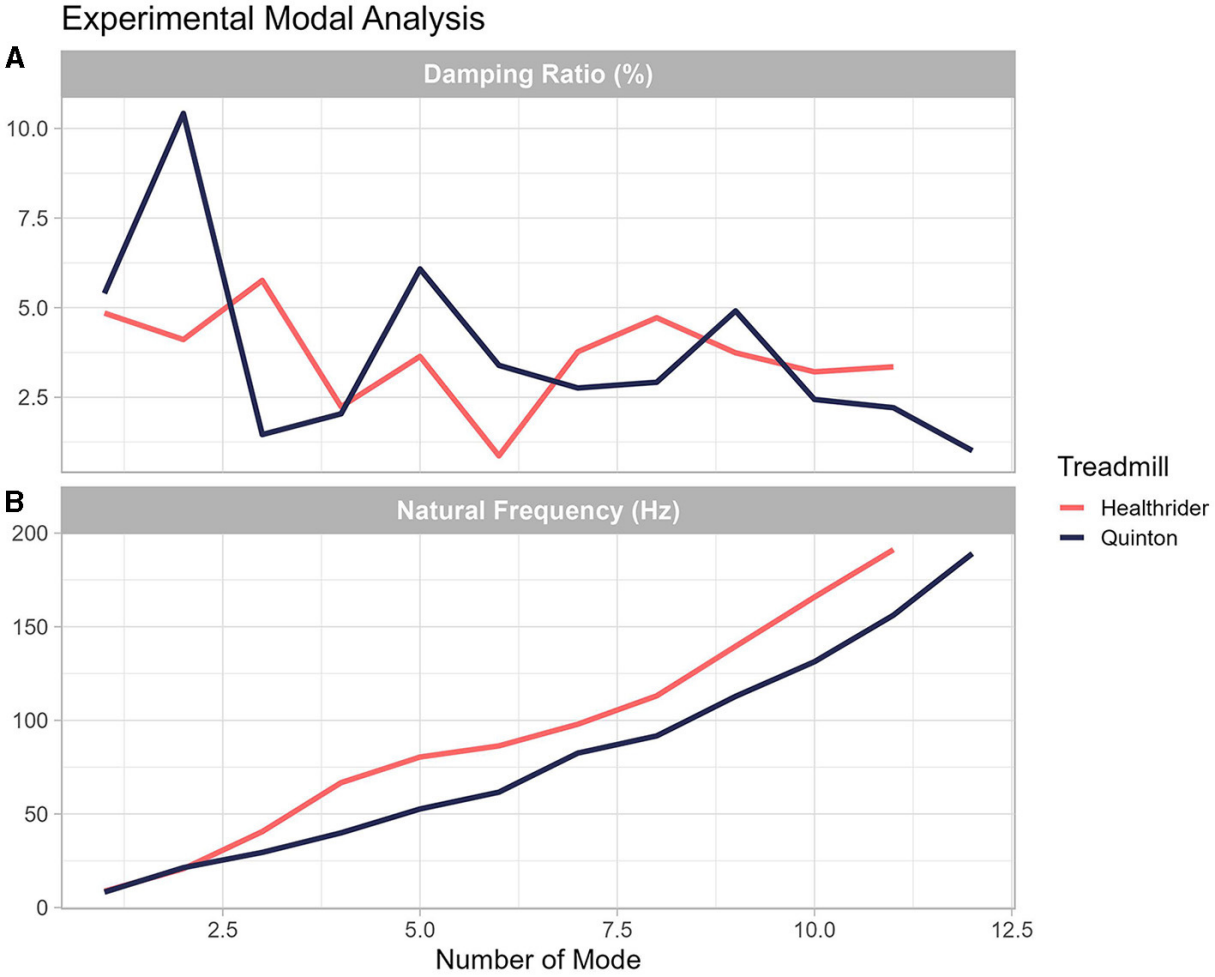
Experimental modal analysis performed on the Healthrider **(A)** and Quinton treadmills **(B)**. The graph shows that the natural frequencies and the damping characteristics of each treadmill are unique.

Differences in the stiffness and damping characteristics in the Quinton and Healthrider treadmill were found by conducting an experimental modal analysis as it can be seen in Figure 5. As seen in Figure 5, the experimental modal analysis provides information of the natural frequency at each mode and the damping ratio between the Quinton and Healthrider treadmill. Descriptively, the amount of damping at each mode is different between the across the different frequencies and at each mode identified, the natural frequency was higher for the Healthrider treadmill.

### 3.4 Variations across strides

Humans inherently express variations in movement patterns. For this reason, we displayed the means and standard deviations of our muscle activity stride measures for each participant in the Supplementary Table 1. This information is presented for each treadmill at each different speed for each muscle.

### 3.5 Summary

Differences in muscle activity were primarily found between the treadmill conditions and overground running, while the only differences between treadmill conditions occurred in the pre-heel strike phase using our second method of separating the muscle activity around heel-strike. All the differences between the different treadmill conditions and overground running produced a medium effect size, except for the biceps femoris muscle activity that showed a large effect size during the stance phase with Method 1.

## 4 Discussion

The primary purpose of the study was to determine if muscle activity was altered when running on different types of treadmills and how muscle activity differed between overground and treadmill running. In line with our first hypothesis, the main findings are that running on different motorized treadmills does affect the muscle activity during the stance phase, swing phase, and immediately before and immediately after heel-strike. In line with our second hypothesis, differences in muscle activity were only observable for one muscle in the pre-heel strike period between the different treadmill conditions.

In terms of the differences between treadmill and overground running, across all speed conditions, muscle activity was greater for the gastrocnemius medialis, biceps femoris, and rectus femoris muscles during overground running relative to treadmill running during the stance phase of the gait cycle. Previous research has suggested that tibialis anterior, biceps femoris and rectus femoris muscle activity is different between treadmill and overground running (Wank et al., 1998; Lee and Hidler, 2008; Wang et al., 2014), while other research has shown no differences in muscle activity between treadmill and overground running (Murray et al., 1985; Arsenault et al., 1986). Our research findings are in line with the research from Wang et al. (2014) such that they showed lower muscle activity for the rectus femoris and biceps femoris during running on a treadmill compared to concrete, rubber and grass surfaces. Additionally, Lee and Hidler (2008) also observed significantly lower activity of the tibialis anterior throughout stance phase during treadmill walking relative to overground walking. One reason for these differences might be because the treadmill belt does allow the leg to be “pulled” under the person's upper body while they run. This attribute could allow the knee flexors to reduce its activity during the stance phase of running. We did in fact see a large effect for the bicep femoris muscle activity such that it was lower during treadmill running vs. overground running. Alternatively, the consistency of running on a treadmill may be different than the laboratory overground conditions of running across a force plate in a 30m runway, allowing runners to move in a predictable manner and reduce muscle activity. Lastly, we did not record from other muscles that control the knee such as the vastus medialis and vastus lateralis. Reductions in muscle activity in one muscle could have resulted in increases in another muscle that we did not record from. Nevertheless, we indicate that muscle activity is lower during treadmill running compared to overground running at least for the key lower limb muscles studied in this present study.

Comparing across the different treadmill conditions, we only observed differences in one muscle in the pre-heel strike period, namely the rectus femoris muscle. It is likely that the treadmill condition has less influence on the muscle activity during the stance phase of running, particularly late stance. During heel-strike, the knee initially moves through flexion followed by extension in the later parts of stance. The rectus femoris is a strong knee flexor and for this muscle, we observed lower muscle activity for the home exercise, Healthrider treadmill. It could be that the rectus femoris muscle was increasing its muscle activity prior to heel-strike for the more research grade Quinton and Bertec treadmills relative to the Healthrider treadmill.

Given that there were differences in muscle activity seen in this study, it begs the question as to why these changes could have occurred. Research supporting changes in muscle activity across treadmills suggested that these differences were due to changes in running kinematics during treadmill running (Nigg et al., 1995). We cannot rule out that the changes in muscle activity can lead to changes in kinematics, however, other researchers have argued that kinematics of overground and treadmill are running same (Riley et al., 2007). In an early study, van Ingen (1980) used a mechanical model with kinetic and potential energy calculations to compare treadmill vs. overground running and concluded that running on a treadmill and overground are similar as long as the treadmill has a constant belt speed. Measuring the belt speed for the treadmills used in this present study showed that the accuracy of the speed was on average 2.2% over all speed settings and at the speed conditions chosen for this study (2.4, 3.6, 4.5 m/s) was an average of 0.6%. Given that the locomotion pattern should be same with a constant belt speed, our study suggests that muscle activity changes during treadmill running in this study and previous studies (Lee and Hidler, 2008) could be attributed to another factor.

One often overlooked factor that could be creating differences in muscles activity is the different mechanical properties of treadmills (Asmussen et al., 2019; Colino et al., 2020), which is important given that different treadmills with potentially different mechanical properties have been used in research studies (Van Hooren et al., 2020). To support this reasoning, differences in treadmill compared to overground running have been observed, specifically, there are subtle changes in the reaction forces (for more information, see Asmussen et al., 2019) that could be creating differences in the muscle activity. The experimental modal analysis (shown in Figure 5) confirmed these differences across running conditions and indicated that each treadmill's natural frequency and damping ratio were unique. These findings support other research that has shown that, across 77 treadmills analyzed, treadmills exhibit different shock absorption, vertical deformation, and energy return (Colino et al., 2020). Several researchers have already shown that humans adjust their leg stiffness to compensate for differences in surface mechanical properties (Ferris et al., 1999; Kerdok et al., 2002). Modifications of leg stiffness are typically driven by muscle activity. Human must adapt to mechanical properties of the surface prior to contact, which can vary across different types of treadmills (e.g., stiff research vs. commercial treadmills), and leads us to conclude that the mechanical properties of the treadmill are an additional factor that are creating changes in muscle activity between treadmill and overground running.

The mechanical properties of an surface is an important determinant of performance and injury risk (Colino et al., 2020) and because mechanical properties differ across treadmill models, this factor should be considered when examining treadmill running. This factor may be even more important to consider when examining how another surface that interacts between the runner's foot and the treadmill, specifically different footwear. Various treadmill models are used by the general public. According to statista, in 2017 the number of users (six years and older) of treadmills is up to 52.97 million (Statista, 2022). Although we have only tested three out of many various models, we did show that muscle activity is altered between treadmills when preparing for heel-strike. However, it still remains unclear how surfaces with comparable stiffness but different damping characteristics affect performance and injury risk (Colino et al., 2020). Therefore, we suggest that further research consider different treadmill models and specifically examine how the mechanical properties may influence the performance, risk of injury, and interaction of footwear with a surface.

As with any study, there are limitations based on our results. There was an allowance of 0.5 m/s for each target speed of each running speed (i.e., 2.7, 3.6, 4.5 m/s). As a percentage, this would allow a different percentage of variation relative to the target speed. On one hand, this allowance could result in a person running at a different speed for the treadmill vs. overground conditions, which is important to note for the interpretation of our results. On the other hand, participants could run slower or faster than the target speed and if participants randomly were equally faster or slower, this allowance may not influence the main muscle activity results (i.e., mean differences). Although we did a power analysis to determine our sample size, we could still have type I errors from our findings because of the smaller sample size, our study design differing than the data we based our power analysis on, and that two participants could not be included in the data analysis. We also used 5 strides to determine each participant's mean value to incorporate into our statistical analyses. Although 5 strides are typically used in biomechanics studies, recent research has indicated that a participant's mean value may not be stable with 5 strides and at a minimum 10 or more strides might be necessary for a given participant (Oliveira and Pirscoveanu, 2021; Yaserifar and Oliveira, 2022). Having only 5 strides could have introduced additional variability in our measures. This in part could explain some variation in our findings of differences across certain running speeds. An example would be that certain muscles showed activity differences at a certain speed such as the tibialis anterior muscle when running at the faster speeds (3.6 m/s, 4.5 m/s), but not the slower speed. This could be attributed to a lack of statistical power or an alternative explanation. One alternative explanation could be that changing the running speed did have subtle changes on running kinematics that could have resulted in different patterns of muscle activity that emerged between treadmill and overground running. We did not record the kinematics because of experimental set-up difficulties and access to treadmills simultaneously with a motion capture system. Lastly, we also recorded from only four muscles of the lower limb and three of which were bi-articular muscles. We chose these muscles to represent muscle activity from muscles that control multiple joints and could be different with our running conditions. Our results and interpretations, however, may have been different if we recorded from other mono-articular muscles such as the vastus medialis, vastus lateralis, soleus, or muscles that primarily control pronation/supination of the foot (e.g., peroneus longus, peroneus brevis, tibialis posterior). Future research could explore the findings from our study and determine if in fact, muscle activity differences across running surfaces is dependent on the running speed or the lower limb muscles that are studied.

To our knowledge, this is the first study which observed muscle activity differences on different types of motorized treadmills that vary in terms of mechanical properties. Although the differences between the different treadmills in our study were minor, we suggest that differences in muscle activity must be considered when comparing treadmill to overground running – at least with the treadmills observed in our study. We showed lower muscle activity in treadmill running vs. overground running for muscles controlling the knee (e.g., biceps femoris, rectus femoris). This finding may be important from a rehabilitation perspective because if a person would like to return to exercise after a knee joint or injury to one of these muscles, treadmill running may be the first activity to pursue before overground running. These findings of muscle activity differences across treadmill and overground running is important because these differences in muscle activity can be further compounded by individuals running on treadmills with larger discrepancies in mechanical properties compared to the treadmills used in this study. Therefore, if one wants to study muscle activity during treadmill running in the stance phase, a commercial treadmill with similar mechanical properties to the treadmills in our study may be sufficient for a research study and implemented instead of an expensive, stiff, high-end research treadmill when running at lower speeds. When comparing muscle activity during treadmill running at higher speeds and prior to heel-strike, a stiff, high-end research treadmill is likely required.

## 5 Conclusion

Our results indicate that running on different types of treadmills with different construction features and mechanical properties does affect the muscle activity compared to overground running and even as early as the preparation for the impact force when running on different treadmills (i.e., before heel strike). Future research should study gait on a variety of other general use treadmills with varying mechanical properties and determine how muscle activity during human locomotion differs across these treadmills. This current research and future lines of inquiry has vast application to the majority of treadmill runners that use common commercial treadmills because, depending on the speed conditions and gait phase, researchers can use these general use, inexpensive treadmills in their studies instead of conducting research relying solely on more stiff research treadmills that (1) may be too expensive for certain research labs to purchase and (2) may have limited ecological validity to the typical treadmill user.

## Data availability statement

The raw data supporting the conclusions of this article will be made available by the authors, without undue reservation.

## Ethics statement

The studies involving humans were approved by University of Calgary Conjoint Health Research Ethics Board. The studies were conducted in accordance with the local legislation and institutional requirements. The participants provided their written informed consent to participate in this study.

## Author contributions

CK: Conceptualization, Data curation, Formal analysis, Methodology, Project administration, Software, Validation, Writing – original draft, Writing – review & editing. AG: Formal analysis, Methodology, Supervision, Writing – review & editing. BN: Conceptualization, Methodology, Project administration, Supervision, Writing – review & editing. MA: Conceptualization, Data curation, Formal analysis, Investigation, Methodology, Project administration, Software, Supervision, Validation, Visualization, Writing – original draft, Writing – review & editing.
