# Supplementary material for: Comparison of muscle activity of the lower limbs while running on different treadmill models
Source: Front Hum Neurosci. 2024 Apr 4;18:1341772. doi: 10.3389/fnhum.2024.1341772 (PMC11024346; doi:10.3389/fnhum.2024.1341772)
Supplement: Supplementary file 1 [file Table_1.docx]

**Supplementary Table** The table represents the mean and standard deviation across the five strides for each subject, each running condition, and each speed for every muscle. Annotation: sd: standard deviation, TA: M. tibialis anterior, GM: M. gastrocnemius medialis, BG: M. biceps femoris, RF: M. rectus femoris.

| **Running**  **condition** | **Speed** | **Muscle** | **Subject 1** | **Subject 2** | **Subject 3** | **Subject 4** | **Subject 5** | **Subject 6** | **Subject 7** | **Subject 8** | **Subject 9** |
| --- | --- | --- | --- | --- | --- | --- | --- | --- | --- | --- | --- |
|  |  |  | mean ± sd | mean ± sd | mean ± sd | mean ± sd | mean ± sd | mean ± sd | mean ± sd | mean ± sd | mean ± sd |
| Bertec | 2.6 m/s | BF | 31.48 ± 6.34 | 20.54 ± 3.29 | 24.27 ± 4.76 | 56.97 ± 12.83 | 57.87 ± 6.2 | 51.98 ± 8.92 | 40.52 ± 7.21 | 25.42 ± 2.62 | 26.48 ± 2.55 |
|  |  | GM | 61.01 ± 6.61 | 44.53 ± 2.36 | 49.24 ± 6.43 | 135.1 ± 15.15 | 60.53 ± 6.87 | 118.44 ± 10.75 | 37.75 ± 3.9 | 64.53 ± 4.59 | 102.95 ± 6.96 |
|  |  | RF | 18.63 ± 2.54 | 37 ± 5.25 | 14.99 ± 1.64 | 51.78 ± 3.53 | 16.66 ± 2.55 | 16.05 ± 2.16 | 43.02 ± 6.13 | 17.98 ± 1.23 | 9.99 ± 0.83 |
|  |  | TA | 88.32 ± 4.72 | 76.72 ± 4.26 | 55.55 ± 5.0 | 36.86 ± 3.91 | 80.08 ± 8.28 | 36.3 ± 4.09 | 150.18 ± 15.81 | 115.47 ± 6.08 | 71.1 ± 3.93 |
|  | 3.6 m/s | BF | 53.21 ± 6.17 | 26.28 ± 5.26 | 35.27 ± 6.27 | 77.66 ± 9.44 | 57.78 ± 7.47 | 73.36 ± 5.36 | 38.71 ± 4.54 | 37.24 ± 4.48 | 36.92 ± 6.76 |
|  |  | GM | 67.29 ± 9.26 | 52.59 ± 3 | 51.19 ± 3.87 | 167.15 ± 10.38 | 59.86 ± 4.9 | 145.51 ± 8.42 | 40.69 ± 2.48 | 81.51 ± 11.84 | 122.74 ± 9.69 |
|  |  | RF | 28.55 ± 2.82 | 48.72 ± 8.3 | 22.09 ± 2.28 | 72.62 ± 6.04 | 15.68 ± 2.77 | 21.42 ± 1.93 | 48.84 ± 4.39 | 22.32 ± 3.76 | 16.42 ± 1.82 |
|  |  | TA | 96.18 ± 3.64 | 88.01 ± 8.61 | 66.35 ± 3.11 | 46.5 ± 5.63 | 80.93 ± 6.78 | 50.85 ± 3.22 | 167.13 ± 28.96 | 134.13 ± 10.77 | 86.66 ± 8.16 |
|  | 4.5 m/s | BF | 68.25 ± 10.63 | 36.15 ± 5.45 | 40.8 ± 3.71 | 70.33 ± 8.45 | 77.28 ± 11.47 | 88.74 ± 7.72 | 43.46 ± 4.33 | 58.89 ± 7.1 | 47.65 ± 6.95 |
|  |  | GM | 81.17 ± 12.42 | 66.88 ± 6.14 | 65.26 ± 5.43 | 174.74 ± 12.74 | 70.94 ± 7.32 | 172.82 ± 13.83 | 45.11 ± 4.07 | 98.52 ± 14.05 | 149.79 ± 22.37 |
|  |  | RF | 31.75 ± 4.1 | 58.45 ± 12.05 | 28.67 ± 3.73 | 77.24 ± 6.87 | 26.02 ± 3.56 | 27.61 ± 2.28 | 61.31 ± 9.7 | 29.84 ± 2.89 | 20.13 ± 1.64 |
|  |  | TA | 109.59 ± 14.7 | 112.89 ± 9.45 | 89.29 ± 8.96 | 51.24 ± 2.96 | 124.08 ± 7.57 | 66.95 ± 3.97 | 190.97 ± 16.15 | 176.92 ± 21.28 | 94.64 ± 7.54 |
| Healthrider | 2.6 m/s | BF | 31.44 ± 5.13 | 23.15 ± 6.6 | 26.87 ± 4.01 | 56.86 ± 9.35 | 61.71 ± 11.05 | 52.38 ± 5.28 | 38.04 ± 6.78 | 29.97 ± 5.39 | 20.57 ± 4.46 |
|  |  | GM | 61.14 ± 8.23 | 56.53 ± 4.5 | 52.04 ± 4.06 | 139.29 ± 19.32 | 74.13 ± 8.52 | 118.71 ± 7.18 | 42.62 ± 4.44 | 72.27 ± 5.91 | 101.08 ± 9.86 |
|  |  | RF | 18.85 ± 1.64 | 39.57 ± 4.79 | 13.59 ± 1.11 | 52.63 ± 7.73 | 18.64 ± 2.18 | 15.27 ± 1.92 | 47.47 ± 5.75 | 17.83 ± 2.31 | 8.32 ± 0.96 |
|  |  | TA | 76.11 ± 7.74 | 76.45 ± 6.04 | 54.24 ± 6.55 | 36.6 ± 5.28 | 73.11 ± 7.6 | 34.28 ± 4.2 | 136.6 ± 18.56 | 96 ± 14.57 | 76.27 ± 9.27 |
|  | 3.6 m/s | BF | 45.64 ± 8.87 | 27.13 ± 4.77 | 36.94 ± 7 | 76.91 ± 10.25 | 71.36 ± 8.22 | 58.34 ± 6.04 | 43.43 ± 6 | 38.99 ± 2.61 | 32.11 ± 4.94 |
|  |  | GM | 71.51 ± 11.58 | 59.04 ± 4.37 | 50.25 ± 5.12 | 167.63 ± 15.58 | 76.7 ± 7.91 | 130.7 ± 6.36 | 48.05 ± 5.17 | 81.74 ± 11.18 | 113.97 ± 17.9 |
|  |  | RF | 27.11 ± 4.6 | 46.53 ± 5.74 | 16.68 ± 1.99 | 67.03 ± 9.93 | 17.64 ± 1.91 | 20.88 ± 2.79 | 52.24 ± 5.4 | 23.1 ± 2.99 | 12.5 ± 1.11 |
|  |  | TA | 87.81 ± 4.86 | 88.83 ± 4.61 | 58.35 ± 3.96 | 50.06 ± 6.61 | 79.39 ± 7.26 | 41.9 ± 2.02 | 154.55 ± 10.75 | 127.64 ± 10.74 | 80.05 ± 9.57 |
|  | 4.5 m/s | BF | 61.49 ± 7.87 | 36.05 ± 9.16 | 35.34 ± 11.02 | 91.42 ± 8.12 | 69.03 ± 15.29 | 69.03 ± 12.53 | 50.74 ± 6.72 | 62.53 ± 6.55 | 35.34 ± 5.87 |
|  |  | GM | 83.42 ± 13.6 | 69.61 ± 9.07 | 60.22 ± 9.1 | 207.25 ± 8.72 | 84.07 ± 6.48 | 150.51 ± 14.08 | 54.6 ± 6.37 | 108.17 ± 13.42 | 144.76 ± 14.09 |
|  |  | RF | 37.87 ± 6.26 | 62.1 ± 9.61 | 25.38 ± 2.47 | 97.98 ± 10.62 | 21.93 ± 2.73 | 20.02 ± 1.24 | 65.66 ± 5.93 | 30.84 ± 6.11 | 16.56 ± 2.32 |
|  |  | TA | 103.31 ± 14.11 | 104.98 ± 8.03 | 73.61 ± 11.58 | 61.21 ± 4.07 | 90.56 ± 8.44 | 51.57 ± 2.08 | 180.39 ± 12.76 | 166.12 ± 13.15 | 93.02 ± 7.83 |
| Quinton | 2.6 m/s | BF | 26.05 ± 3.3 | 21.67 ± 5.14 | 22.91 ± 5.04 | 54.05 ± 6.79 | 39.9 ± 8.85 | 53.04 ± 7.81 | 31.16 ± 4.77 | 45.42 ± 4.85 | 22.52 ± 4.2 |
|  |  | GM | 60.94 ± 1.97 | 50.58 ± 4.73 | 42.96 ± 3.89 | 135.85 ± 17.51 | 64.7 ± 14.62 | 102.48 ± 14.91 | 35.21 ± 4.16 | 84.17 ± 5.16 | 116.2 ± 8.17 |
|  |  | RF | 17.91 ± 1.17 | 34.64 ± 4.61 | 12.67 ± 0.83 | 46.46 ± 3.89 | 22.48 ± 5.03 | 16.55 ± 1.66 | 42.12 ± 4.88 | 22.88 ± 4.84 | 9.02 ± 0.75 |
|  |  | TA | 75.99 ± 6.86 | 87.47 ± 3.86 | 48.11 ± 3.32 | 41.76 ± 4.81 | 104.65 ± 25.84 | 39.88 ± 4.7 | 137.92 ± 17.51 | 89.15 ± 5.44 | 79.83 ± 9.88 |
|  | 3.6 m/s | BF | 44.66 ± 8.95 | 31.15 ± 4.27 | 31.56 ± 4.04 | 87.29 ± 14.93 | 64.76 ± 15.15 | 69.54 ± 3.96 | 33.8 ± 5.49 | 47.01 ± 4.39 | 34.07 ± 6.92 |
|  |  | GM | 73.96 ± 8.78 | 68.81 ± 9.83 | 50.68 ± 5.14 | 160.13 ± 14.73 | 66.28 ± 7.09 | 140.28 ± 14.78 | 44.15 ± 3.6 | 75.37 ± 5.1 | 135.79 ± 27.18 |
|  |  | RF | 24.04 ± 3.26 | 48.63 ± 7.72 | 18.78 ± 1.95 | 72.89 ± 9.03 | 18.08 ± 1.83 | 19.28 ± 2.56 | 47.37 ± 5.18 | 28.48 ± 2.89 | 13.55 ± 2.25 |
|  |  | TA | 80.89 ± 10.75 | 104.48 ± 6.47 | 54.81 ± 3.45 | 50.22 ± 6.32 | 85.4 ± 9.6 | 46.12 ± 2.76 | 152.38 ± 14.97 | 114.96 ± 6.03 | 84.83 ± 6.75 |
|  | 4.5 m/s | BF | 51.58 ± 8.51 | 32.82 ± 4.37 | 35.97 ± 5.12 | 102.86 ± 12.36 | 73.71 ± 12.59 | 76.43 ± 9.1 | 41.25 ± 6 | 63.79 ± 4.41 | 39.36 ± 4.03 |
|  |  | GM | 76.63 ± 11.14 | 72.16 ± 7.17 | 56.11 ± 6.45 | 201.42 ± 16.5 | 66.92 ± 8.08 | 155.64 ± 10.49 | 47.08 ± 4.61 | 77.51 ± 6.36 | 154.2 ± 17.22 |
|  |  | RF | 26.49 ± 4.41 | 65.48 ± 7.22 | 25.2 ± 2.28 | 98.04 ± 8.73 | 25.04 ± 4.26 | 22.17 ± 2.59 | 59.15 ± 4.01 | 26.67 ± 3.04 | 16.75 ± 2.56 |
|  |  | TA | 92.73 ± 16.6 | 125.9 ± 7.81 | 64.24 ± 5.25 | 60.49 ± 3.88 | 109.21 ± 10.08 | 50.58 ± 3.49 | 168.39 ± 15.09 | 136.63 ± 19.54 | 93.52 ± 7.17 |
| Overground | 2.6 m/s | BF | 9.17 ± 1.72 | 5.13 ± 0.9 | 7.36 ± 1.28 | 13.74 ± 3.22 | 14.26 ± 3.9 | 11.08 ± 1.85 | 7.98 ± 2.12 | 7.7 ± 0.66 | 6.05 ± 1.57 |
|  |  | GM | 16.76 ± 1.28 | 14.09 ± 2.52 | 14.46 ± 1.07 | 39.58 ± 4.56 | 16.2 ± 2.76 | 31.11 ± 3.45 | 10.76 ± 0.78 | 20.46 ± 3.75 | 33.88 ± 6.85 |
|  |  | RF | 6.37 ± 0.66 | 9.02 ± 1.85 | 3.6 ± 0.58 | 13.55 ± 2.01 | 5.22 ± 0.64 | 3.78 ± 0.62 | 15.63 ± 1.85 | 3.88 ± 0.86 | 2.89 ± 0.54 |
|  |  | TA | 27.75 ± 2.22 | 17.2 ± 1.59 | 15.08 ± 0.68 | 10.24 ± 1.36 | 22.16 ± 1.88 | 10.03 ± 1.25 | 41.94 ± 6.34 | 24.4 ± 3.76 | 19.01 ± 3.18 |
|  | 3.6 m/s | BF | 12.01 ± 1.32 | 5.68 ± 1.02 | 9.06 ± 1.97 | 20.61 ± 4.44 | 20.34 ± 2.79 | 13.56 ± 2.84 | 10.01 ± 2.3 | 12.88 ± 2.28 | 7.25 ± 3.14 |
|  |  | GM | 22.76 ± 2.93 | 17.86 ± 2.26 | 15.95 ± 1.98 | 54.54 ± 9.59 | 21.17 ± 2.66 | 36.73 ± 4.82 | 13.37 ± 1.84 | 19.43 ± 2.01 | 40.23 ± 16.64 |
|  |  | RF | 8.7 ± 1.45 | 11.52 ± 2.1 | 5.47 ± 1.02 | 19.15 ± 2.94 | 6.37 ± 0.9 | 4.72 ± 0.89 | 19.13 ± 3.04 | 5.33 ± 1.7 | 3.2 ± 0.94 |
|  |  | TA | 24.13 ± 3.84 | 18.37 ± 1.85 | 16.78 ± 1.78 | 12.92 ± 1.03 | 20.4 ± 2.59 | 13.06 ± 1.57 | 41.53 ± 3.72 | 26.45 ± 3.84 | 18.05 ± 6.35 |
|  | 4.5 m/s | BF | 16.5 ± 2.19 | 9.44 ± 1.61 | 11.92 ± 3.17 | 30 ± 4.14 | 21.41 ± 4.79 | 18.01 ± 3.1 | 11.48 ± 1.2 | 17.06 ± 1.55 | 8.84 ± 0.97 |
|  |  | GM | 25.6 ± 2.14 | 20.81 ± 2.53 | 18.63 ± 2.73 | 67.45 ± 8.8 | 22.69 ± 1.42 | 41.07 ± 3.53 | 13.24 ± 0.98 | 25.21 ± 4.25 | 38.29 ± 5.67 |
|  |  | RF | 10.67 ± 1.62 | 15.22 ± 2.84 | 6.48 ± 0.9 | 26.6 ± 4.38 | 8.02 ± 1.12 | 5.69 ± 0.83 | 21.27 ± 2.79 | 6.15 ± 0.75 | 3.14 ± 0.36 |
|  |  | TA | 24.11 ± 3.14 | 23.2 ± 3.05 | 18.08 ± 2.8 | 14.7 ± 1.59 | 27.33 ± 1.83 | 12.89 ± 1.62 | 36.37 ± 4.65 | 31.52 ± 3.42 | 20.22 ± 3.06 |
